# Bitter Taste Receptor Agonists Mitigate Features of Allergic Asthma in Mice

**DOI:** 10.1038/srep46166

**Published:** 2017-04-11

**Authors:** Pawan Sharma, Roslyn Yi, Ajay P. Nayak, Nadan Wang, Francesca Tang, Morgan J. Knight, Shi Pan, Brian Oliver, Deepak A. Deshpande

**Affiliations:** 1 Department of Medicine, Center for Translational Medicine, Thomas Jefferson University, Philadelphia, PA, 19107, USA; 2 Respiratory Research Group, Woolcock Institute of Medical Research and School of Life Sciences, University of Technology, Sydney, NSW, 2007, Australia; 3 Department of Experimental Therapeutics, University of Texas MD Anderson Cancer Center, Houston, TX, 77054, USA

## Abstract

Asthma is characterized by airway inflammation, mucus secretion, remodeling and hyperresponsiveness (AHR). Recent research has established the bronchodilatory effect of bitter taste receptor (TAS2R) agonists in various models. Comprehensive pre-clinical studies aimed at establishing effectiveness of TAS2R agonists in disease models are lacking. Here we aimed to determine the effect of TAS2R agonists on features of asthma. Further, we elucidated a mechanism by which TAS2R agonists mitigate features of asthma. Asthma was induced in mice using intranasal house dust mite or aerosol ova-albumin challenge, and chloroquine or quinine were tested in both prophylactic and treatment models. Allergen challenge resulted in airway inflammation as evidenced by increased immune cells infiltration and release of cytokines and chemokines in the lungs, which were significantly attenuated in TAS2R agonists treated mice. TAS2R agonists attenuated features of airway remodeling including smooth muscle mass, extracellular matrix deposition and pro-fibrotic signaling, and also prevented mucus accumulation and development of AHR in mice. Mechanistic studies using human neutrophils demonstrated that inhibition of immune cell chemotaxis is a key mechanism by which TAS2R agonists blocked allergic airway inflammation and exerted anti-asthma effects. Our comprehensive studies establish the effectiveness of TAS2R agonists in mitigating multiple features of allergic asthma.

Asthma is a chronic inflammatory disease of the airways affecting over 300 million people worldwide^1^ and characterized by airway inflammation, hyperresponsiveness, mucus hypersecretion and remodeling^2^,^3^,^4^. The chronic and persistent inflammation leads to structural and functional changes in the resident airway cells^4^,^5^,^6^,^7^. G protein-coupled receptors (GPCRs) play a vital role in the regulation of airway inflammation, and airway smooth muscle (ASM) contraction, relaxation, and proliferation^8^,^9^. Therefore, GPCR agonists or antagonists comprise mainstay asthma therapies. Identifying a novel class of GPCRs is one of the approaches to developing newer and effective anti-asthma drugs.

We recently identified expression of bitter taste receptors (TAS2Rs) on human ASM and demonstrated that stimulation with TAS2R agonists results in relaxation of ASM^10^. Other laboratories confirmed the expression of TAS2Rs and the bronchodilatory effect of TAS2R agonists in mouse^11^,^12^, human^13^,^14^,^15^ and guinea pig^16^ airways. Administration of TAS2R agonists into the mouse lungs results in bronchodilation in both naïve and allergen-exposed animals^10^, and TAS2R expression, signaling, and ASM relaxation and bronchodilatory effects are maintained under inflammatory conditions in human asthmatic ASM cells and intact lung slices^17^. TAS2Rs have thus emerged as novel targets in the treatment of obstructive airway diseases such as asthma. Numerous other properties of TAS2Rs including the availability of well-established natural and synthetic agonists make TAS2Rs an attractive target (reviewed in refs 18 and 19).

Asthma is a complex disease involving multiple pathogenic features including inflammation, mucus secretion, remodeling and bronchoconstriction. We have previously shown that TAS2R agonists are potent bronchodilators, yet their effect on key features of asthma remains undetermined. Because beta-agonists exhibit little or no anti-inflammatory activity, and no current asthma drugs appear effective in deterring airway remodeling, a “multi-tasking” asthma drug capable of inhibiting bronchoconstriction, airway inflammation, and airway remodeling would constitute a novel therapeutic addressing an important, unmet clinical need. Here we used two different mouse models of allergic asthma to assess the effect of TAS2R agonists on key features of asthma. Our results demonstrate that TAS2R agonists are effective in not only reducing AHR, but also in preventing allergic airway inflammation and multiple features of airway remodeling.

## Results

**Figure 1:**
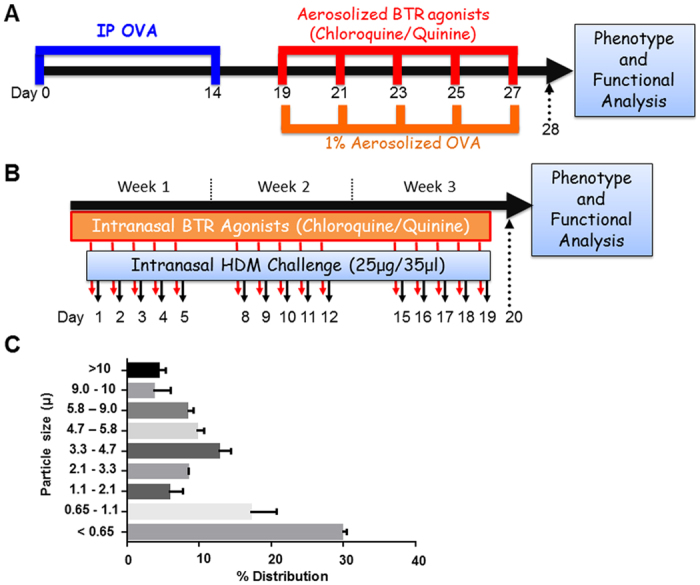
Allergen sensitization and challenge protocols in mice. (**A**) Male FVB/N mice (8-weeks) were injected with 2 mg alum in 0.2 ml PBS or alum with 100 mg ovalbumin (OVA) per mouse on day 1 and day 14 intraperitoneally. On days 19, 21, 23, 25 and 27 mice were challenged with aerosolized OVA (1%) or saline for 30 min. A select set of animals were treated with nebulized chloroquine or quinine (1.5 mg/ml) or vehicle for 30 min prior to OVA challenge each day. (**B**) Female BALB/c mice were challenged with house dust mite (HDM, 25 μg/35 μl) allergen intranasally as shown in the above schematic: 5 days/week for 3 weeks. 30 min prior to HDM challenges, a select set of mice were treated with chloroquine (50 mg/kg) or quinine (10 mg/kg) intranasally. 24 h after the last allergen challenge, phenotype was assessed by collecting lung lavage, formalin-fixed or frozen lung samples, and measuring lung function by flexiVent. Key features of asthma namely airway inflammation, remodeling and hyperresponsiveness were assessed respectively. (**C**) Chloroquine aerosol particle size distribution. Aerosolized Chloroquine (1.5 mg/ml) was captured in an Anderson Cascade Impactor and quantified by HPLC-MS. Based on the aerosol quantification, each animal received 0.5 μg per day based on 30 min of aerosol treatment.

Mice were pretreated with TAS2R agonists, chloroquine or quinine by intranasal (HDM model) or aerosol (OVA model) route, and challenged with either HDM or OVA as described in Methods and illustrated in Fig. 1. Twenty-four hours post-final challenge, lung function, airway inflammation, and features of airway remodeling were assessed. Aerosol characterization of chloroquine showed more than 60% of aerosolized drug was contained in particles of sufficient size (<3.3 μm) to reach and deposit in the lower airways (Fig. 1C)^20^. Aerosolization of a 1.5 mg/ml chloroquine solution produced a mean aerosol concentration of 2.6 μg/L, which corresponds to a 500 ng dose per 30 minutes treatment.

### Effect of TAS2R agonists on allergic airway inflammation

**Figure 2:**
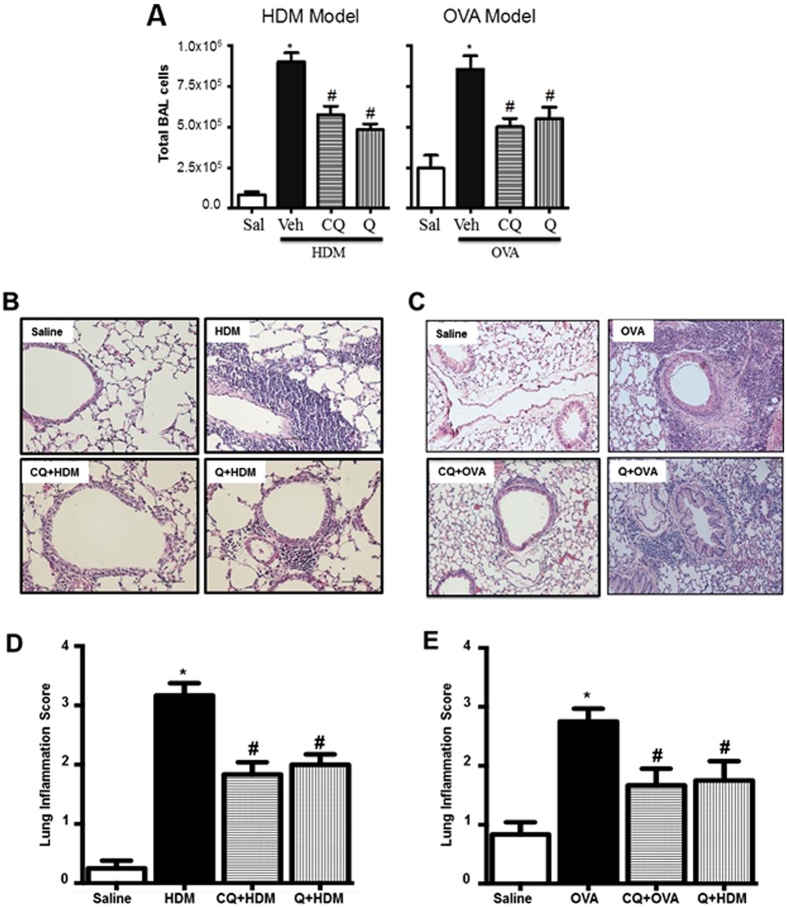
TAS2R agonists inhibit allergen-induced airway inflammation. (**A**) Total BAL cell count was performed using a hemocytometer. Histology was performed on formalin-fixed lungs and then stained with H&E for assessing tissue inflammation (**B**,**C**). Lung inflammation scoring was performed on H&E stained tissue slides as described in the methods section using multiple images (3 images/mouse) (**D**,**E**). Data shown above represent the mean of 8–11 animals in each group; *p < 0.05 (Saline vs HDM or OVA), ^#^*p* < 0.05 (HDM or OVA vs CQ or Q). CQ: chloroquine; Q: quinine.

HDM and OVA challenge resulted in a robust increase in immune cell infiltration in the airways as demonstrated by a significant increase in cell count in the BAL compared to controls. Pretreating mice with TAS2R agonists chloroquine or quinine significantly (*p* < 0.05) reduced infiltration of inflammatory cells (Fig. 2A). This was further confirmed by histological evaluation of H&E stained lung sections (Fig. 2B–E). Differential cell count revealed infiltration of eosinophils, neutrophils and to a lesser extent accumulation of lymphocytes and macrophages in the BAL. TAS2R agonists significantly (*p* < 0.05) inhibited infiltration of eosinophils, neutrophils and macrophages (Table 1). HDM and OVA challenge also caused a significant increase in multiple cytokines and chemokines in the lung, and TAS2R agonists inhibited this induction to different degrees (Table 2). HDM exposure in mice specifically led to significant increases in the levels of pro-eosinophilic-Th2 cytokines (IL-4, IL-5, IL-9 and IL-13), pro-inflammatory cytokines eotaxin, KC, TNF-α, IP-10 and RANTES as well as IL-17, a monocyte and neutrophil chemo-attractant to the tissue and the anti-inflammatory cytokine IL-10. TAS2R agonists differentially inhibited the induction of most cytokines and chemokines, with quinine being more effective than chloroquine in global suppression of inflammation.

In OVA-exposed mice there was a significant induction of IL-4, IL-5, IL-13, IL-17, eotaxin, KC, all of which were significantly suppressed by TAS2R agonists pretreatment (Table 2). Similarly OVA induced TNF-α, IP-10 and RANTES, yet TAS2R agonist pretreatment failed to significantly repress the induction of these agents. Collectively, these results indicate that TAS2R agonists demonstrate anti-inflammatory activity in the lung, albeit with differential efficacy with respect to specific cytokine suppression and the allergen employed.

### Effect of TAS2R agonists on features of airway remodeling

**Figure 3:**
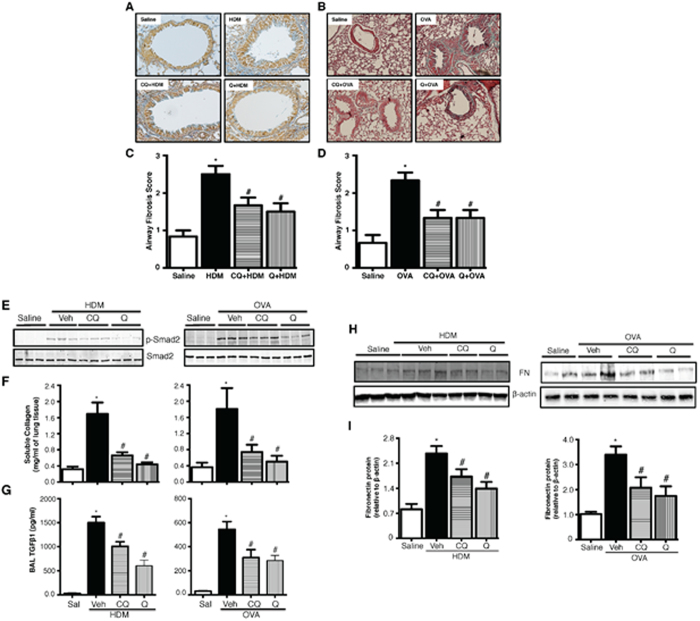
TAS2R agonists mitigate features of fibrotic airway remodeling. Formalin-fixed lungs were stained with AFOG (**A**) or Trichrome (**B**) for assessing airway fibrosis to HDM and OVA. Airway fibrosis scoring was performed as described in the methods for both HDM (**C**) and OVA (**D**) models. Immunoblotting was performed using the lung lysates to assess pro-fibrotic signaling for p-Smad-2 (**E**), Fibronectin (**H**) and β-actin was used as a loading control (**I**). Total soluble collagen (**F**) was also quantified using a Sircol-collagen assay and levels of TGF-β1 (**G**) release in the BAL fluid was measured using multiplex array as described in the methods. Data shown above represent the mean of 8–11 animals in each group; *p < 0.05 (Saline vs HDM or OVA), ^#^*p* < 0.05 (HDM or OVA vs CQ or Q) using one way ANOVA. HDM: house dust mite; OVA: ovalbumin; CQ: chloroquine; Q: quinine, FN: fibronectin.

The effect of TAS2R agonists on allergen-induced airway remodeling was assessed by determining expression of marker proteins in the BAL or lung lysates and by histology. Collagen deposition as assessed by trichrome staining of lung sections was significantly lower in lungs of mice treated with TAS2R agonists (Fig. 3A–D). Secreted collagen was significantly higher in allergen-challenged animals, and chloroquine or quinine pretreatment significantly (*p* < 0.05) inhibited this response (Fig. 3F). Allergen-induced expression of TGF-β1 (a marker of pro-fibrotic remodeling) was markedly inhibited by chloroquine and quinine (Fig. 3G). TGF-β action on airway cells involves phosphorylation of Smad2, and immunoblot analysis of lung lysates revealed inhibition of allergen-induced increase in phospho-Smad2 by TAS2R agonists (Fig. 3E). Finally, immunoblot analyses of whole lung lysates revealed increased expression of fibronectin (Fig. 3H,I) in HDM- or OVA-exposed mice, which was inhibited by pretreatment with TAS2R agonists.

**Figure 4:**
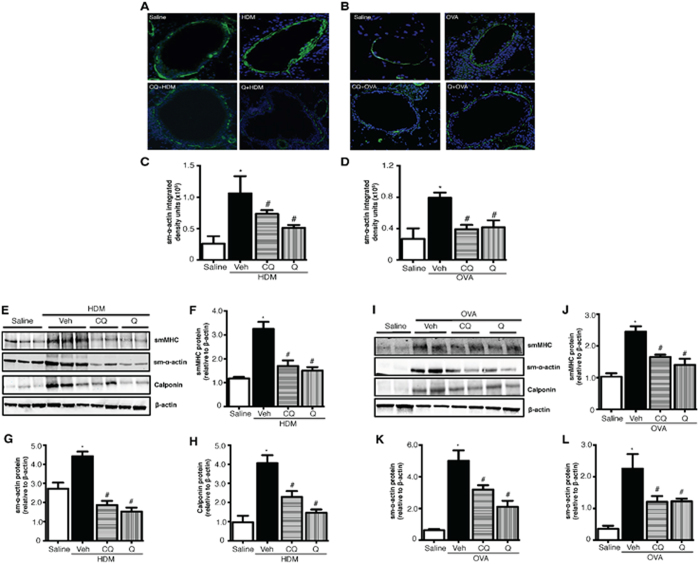
TAS2R agonists inhibit accumulation of smooth muscle mass upon allergen challenge. Airway smooth muscle mass was determined by immunofluorescence method in both HDM (**A**) and OVA (**B**) models. Stained images were then quantified using Image J analysis software to determine sm-α-actin staining in the lungs (**C**,**D**). Key smooth muscle marker proteins were assessed by immunoblotting using the lung-lysates in the HDM model. Representative western blots (**E**) and densitometric analyses for smMHC (**F**), sm**-**α-actin (**G**) and calponin (**H**) are shown in the figure. Similarly smooth muscle markers were assessed in the OVA model as shown in the representative immunoblots (**I**) with densitometric analysis for smMHC (**J**), sm**-**α-actin (K) and calponin (**L**). β-actin was used as a loading control. Data shown above represent the mean of 8–11 animals in each group; *p < 0.05 (Saline vs HDM or OVA), ^#^*p* < 0.05 (HDM or OVA vs CQ or Q) using 1-way ANOVA. HDM: house dust mite; OVA: ovalbumin; CQ: chloroquine; Q: quinine.

Immunofluorescence staining of sm-α-actin in lung sections revealed increased smooth muscle around the airways in HDM or OVA challenged animals, and TAS2R agonists significantly (*p* < 0.05) inhibited this induction (Fig. 4A–D). Moreover, the accompanying allergen-induced increase in smMHC, sm-α-actin, and calponin expression in lung lysates was similarly inhibited by chloroquine and quinine pretreatment in HDM (Fig. 4E–H) and OVA (Fig. 4I–L) models of allergic asthma.

**Figure 5:**
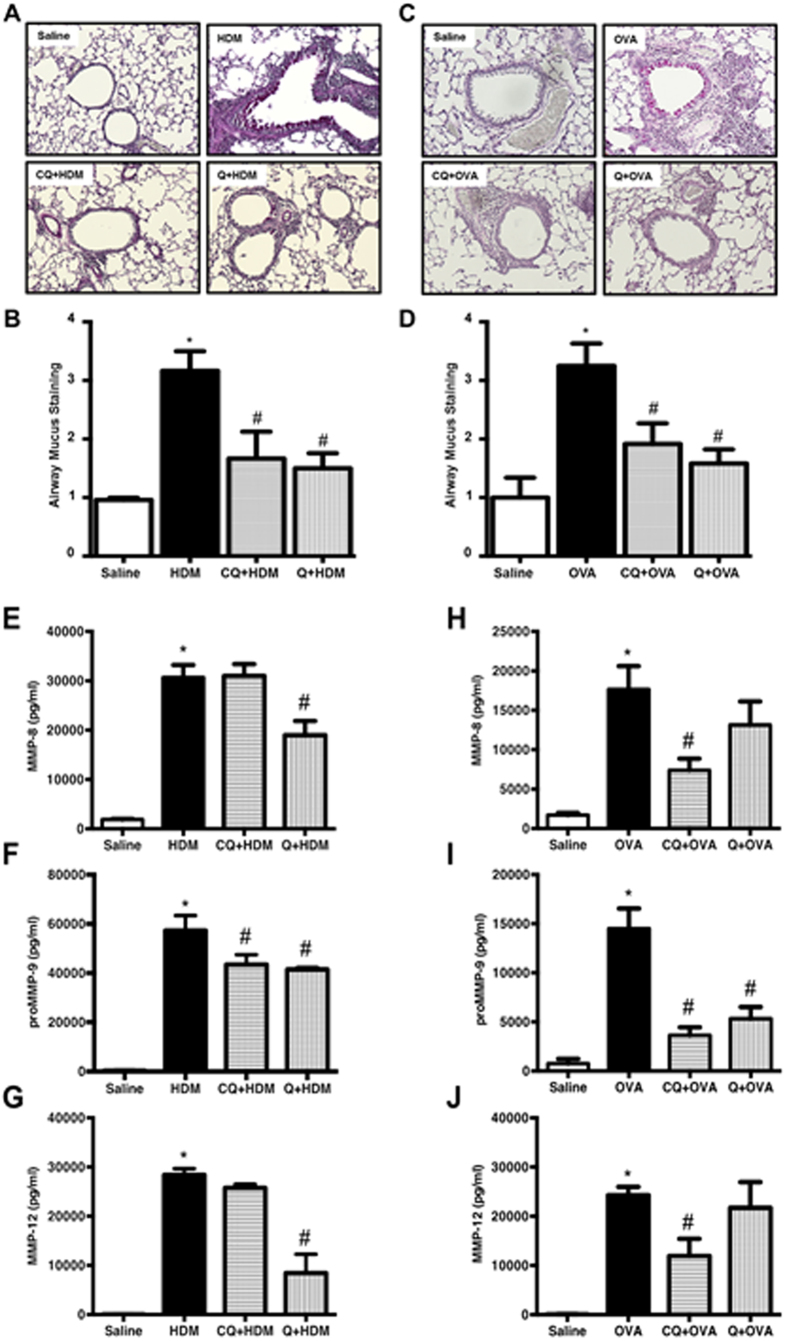
TAS2R agonists inhibit mucus accumulation and MMP release in the airways. Formalin-fixed airways were stained with PAS to assess mucus accumulation in the airways in both HDM model (**A**) and OVA model (**C**). Airway mucus staining was blindly graded using multiple images (3 images/mouse) for both HDM (**B**) and OVA (**D**). A variety of MMPs were measured in the BAL namely MMP-8 (**E**,**H**), proMMP-9 (**F**,**I**) and MMP-12 (**G**,**J**) in HDM and OVA animals respectively. Results are described as pg/ml. Data shown above represents the mean of 8–11 animals in each group; *p < 0.05 (Saline vs HDM or OVA), ^#^*p* < 0.05 (HDM or OVA vs CQ or Q) using 1-way ANOVA. CQ: chloroquine; Q: quinine.

Increased mucus accumulation is another key feature of asthma. HDM or OVA challenge resulted in excessive accumulation of mucus in the airways (assessed by PAS staining), and pre-treatment with TAS2R agonists inhibited this accumulation (Fig. 5A–D). Matrix metalloproteinases (MMPs) have been implicated in asthmatic airway remodeling. HDM or OVA challenge resulted in robust induction of MMP-8 (neutrophil collagenase), pro-MMP-9 (gelatinase) and MMP-12 (macrophage metalloelastase) in BAL fluid, when compared to saline-challenged mice (Fig. 5E–J). In the HDM model, TAS2R agonist quinine was more effective than chloroquine in blocking induction of MMP-8 and MMP-12 while both TAS2R agonists blocked pro-MMP-9 induction. In the OVA model, chloroquine was more effective than quinine in blocking induction of MMP-8 and MMP-12, whereas both TAS2R agonists blocked pro-MMP-9 induction. These results suggest that TAS2R agonists differentially regulate MMPs induction in models of allergic asthma.

### Effect of TAS2R agonists on airway hyperresponsiveness

**Figure 6:**
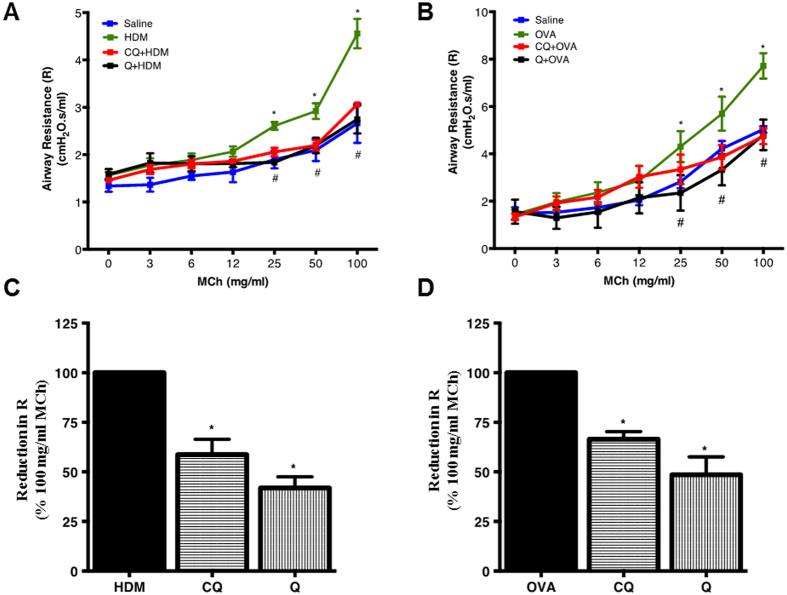
Allergen-induced AHR is diminished upon pre-treatment with TAS2R agonists. Mice were subjected to an increased dose of nebulized methacholine (MCh) challenge protocol to assess characteristics of respiratory mechanics in HDM (**A**) and OVA model (**B)**. Animals were treated with aerosolized chloroquine or quinine at the end of the highest concentration of MCh and % change in R was computed in HDM (**C**) or OVA (**D**) group. *P < 0.05, was considered significant for HDM or OVA *vs* saline and ^#^*P* < 0.05, was considered significant for HDM or OVA *vs* CQ or Q treated group. Data shown is the mean of 8-11 mice in each group.

Airway responsiveness to MCh challenge was determined using anesthetized, intubated and ventilated mice. HDM challenge resulted in an increased bronchoconstrictor response to MCh, and pretreatment with TAS2R agonists inhibited development of AHR (Fig. 6A). A similar but slightly increased bronchoconstrictor response occurred in OVA-challenged mice, and again this was reversed by pretreatment with chloroquine or quinine (Fig. 6B). Furthermore, aerosolized chloroquine (250 μg/ml) and quinine (150 μg/ml) (administered after the highest concentration of MCh) were able to provide effective bronchorelaxation as seen by significant reduction in airway resistance (R) in both HDM- and OVA- challenged mice (Fig. 6C,D).

### Effect of TAS2R agonists on allergic airway inflammation in treatment model

**Figure 7:**
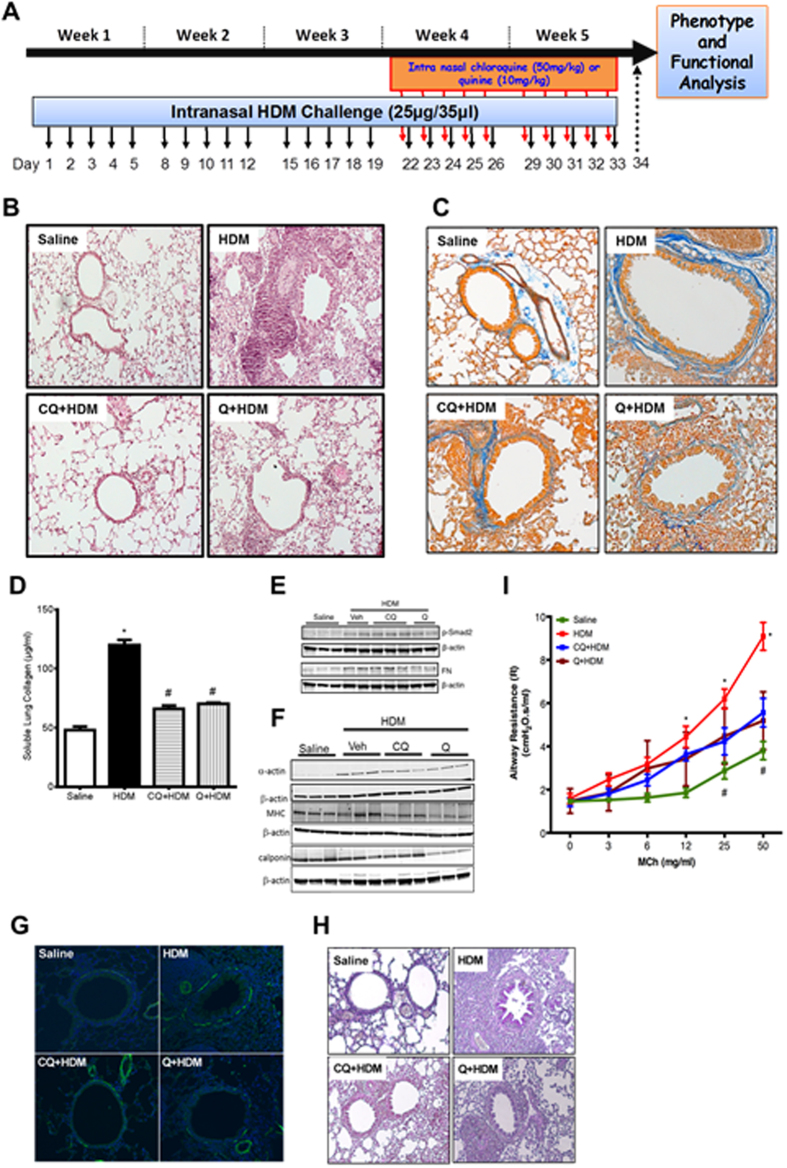
Effect of TAS2R agonists on features of allergic asthma in the treatment model. (**A**) Female BALB/c mice were initially dosed 5 days a week for 3 weeks with 25 μg of HDM extract. Mice were then treated with either chloroquine (CQ, 50 mg/kg) or quinine (Q, 10 mg/kg) or vehicle (1% ethanol) 30 min prior to subsequent HDM challenges for additional 2 weeks. (**B**) Histopathological assessment of tissue inflammation was performed on formalin-fixed lung tissue using H&E staining. (**C**) Formalin-fixed lungs were stained with Trichrome for assessing airway fibrosis to HDM. (**D**) Total soluble collagen in lung tissue was quantified using a Sircol-collagen assay. (**E**) Immunoblotting was performed using the lung lysates to evaluate pro-fibrotic signaling for p-Smad-2 and fibronectin (FN). (**F**) Representative western blots for sm**-**α-actin, smMHC, and calponin are shown. β-actin was used as a loading control. (**G**) ASM mass was determined by staining formalin-fixed lung tissue for sm-α-actin using immunofluorescence method. (**H**) Fixed lungs were stained with PAS to assess accumulation of mucus producing cells in the bronchioles. (**I**) Mice were subjected to an increased dose of nebulized methacholine (MCh) challenge protocol to assess features of respiratory mechanics. *P < 0.05, was considered significant for HDM or OVA *vs* saline and ^#^*P* < 0.05, was considered significant for HDM or OVA *vs* CQ or Q treated group. Data shown is the mean of 8–11 mice in each group.

The ability of TAS2R agonists in attenuating various features of allergic airway inflammation was further evaluated in a treatment model. Allergic inflammation was established in mice *via* intranasal administration of HDM for 3 weeks and following this, for next 2 weeks mice were treated with TAS2R agonists, chloroquine or quinine by intranasal route, and challenged with HDM (Fig. 7A). Treatment of mice with TAS2R agonists resulted in reduced infiltration of the inflammatory cells in the airways and attenuation of histopathological changes in the lung tissue (Fig. 7B and C). Significant reductions were observed in accumulation of eosinophils, neutrophils and to lesser extent macrophages in the airways following treatment with TAS2R agonists (Table 1). Furthermore, treatment with TAS2R agonists resulted in significant reduction in levels of allergen-induced cytokines such as eotaxin, KC, IP-10 and RANTES in the airways (Table 3). Repeated instillation of HDM in the airways for 5 weeks resulted is extensive collagen deposition around the conducting airways (Fig. 7C), which was attenuated by treatment with TAS2R agonists. Furthermore, soluble collagen levels in lung tissue lysates were also significantly lower following treatment with TAS2R agonists (Fig. 7D). Pro-fibrotic cytokine TGF-β1 expression was prominent in mice exposed with HDM and its expression was attenuated in mice treated with TAS2R agonists (Table 3). In concert, we also observed inhibition of HDM-induced accumulation of phospho-Smad2 as well as fibronectin levels by treatment with TAS2R agonists (Fig. 7E). Treatment of mice with quinine also resulted in reduced expression of sm-α-actin, smMHC and calponin in the lung tissue compared to untreated mice (Fig. 7F and G). Comparable effects were observed following treatment with chloroquine, although calponin expression was not affected. Treatment with TAS2R agonists also resulted in fewer PAS^**+**^ mucus-producing cells in the bronchi and bronchioles (Fig. 7H). Finally, treatment with TAS2R agonists resulted inhibition of dose-dependent MCh-induced bronchoconstrictor response in HDM-challenged mice (Fig. 7I).

### Mechanism of action of TAS2R agonists

**Figure 8:**
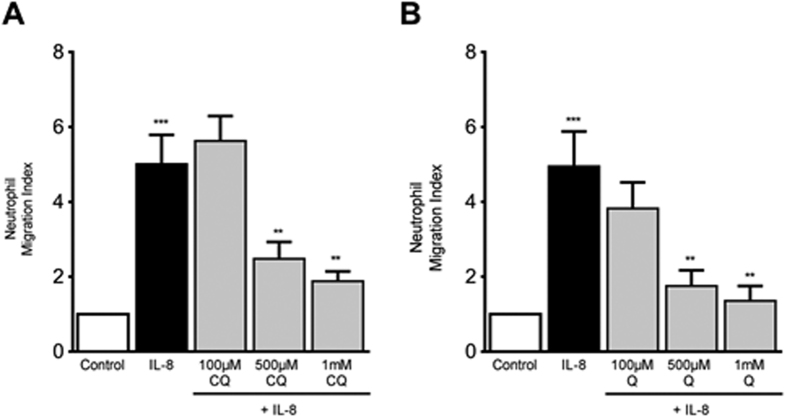
Effect of TAS2R agonists on neutrophil chemotaxis. The neutrophil chemotaxis or migration to CXCL8 was measured using Transwell inserts. Isolated and purified peripheral human blood neutrophils were plated at desired density in the upper chamber of the Transwell with or without TAS2R agonists (CQ and Q) at increasing concentrations (100 μM, 500 μM and 1 mM) for 90 minutes at 37 °C while a chemotactic gradient was created using IL-8 (100 ng/ml) in the lower chamber of the Transwell plate. The results are expressed as fold change in cell migration from upper well to lower well and expressed as neutrophil migration index relative to control, which is kept as 1. Data shown above represent the mean of 5-human donors in each group; *p < 0.05 (Control *vs* IL-8), ^#^*p* < 0.05 (IL-8 *vs* CQ or Q) using 1-way ANOVA. CQ: chloroquine; Q:quinine.

As TAS2R receptor agonists exhibited protective effects on airway inflammation through attenuated expression of cytokines and chemokines and significant reduction in allergen-induced immune cell influx into the mouse lungs, we investigated the potential mechanism for the broad anti-inflammatory action of TAS2R agonists. Using peripheral blood neutrophils from healthy human volunteers we studied the effect of TAS2R agonists on CXCL8 (IL-8)-mediated chemotaxis of immune cells (neutrophils) (Fig. 8). Our results demonstrate dose-dependent reduction in the immune cell recruitment to a chemotactic gradient (CXCL8, 100 ng/ml, 90 min) by both TAS2R agonists chloroquine and quinine; the effect being more pronounced and significant at 500 μM and 1 mM respectively.

Our previous study using human ASM cells suggest that TAS2R agonists inhibit mitogen-induced ASM growth^21^. The anti-remodeling effect of TAS2R agonists observed in this study may involve anti-mitogenic effect of TAS2R agonists. TAS2R agonists induce efficacious bronchodilation in murine models^10^,^11^,^12^ and human ASM cells/tissues. The bronchodilatory effect may account for an attenuated AHR in animals treated with TAS2R agonists compared to allergen-challenged animals.

## Discussion

Recent studies have established the expression of TAS2R on extraoral tissues including ASM. Most importantly, stimulation of airways with a variety of TAS2R agonists results in relaxation of airways and bronchodilation. Based on this observation TAS2Rs have emerged as novel, promising targets for treating obstructive lung diseases such as asthma. To further validate the potential use of TAS2Rs as anti-asthma drug targets, we assessed the effectiveness of two different bitter ligands (chloroquine and quinine) delivered by two different routes (intranasal and aerosol) in murine models challenged with two different allergens (OVA and HDM). Aerosol quantification further confirmed the feasibility of delivering chloroquine to lower airways by aerosol route. Our findings suggest that TAS2R agonists mitigate allergen-induced airway inflammation, remodeling, mucus secretion and AHR in murine models of asthma.

TAS2Rs are known to be activated by a variety of structurally diverse natural and synthetic compounds. Most of the TAS2Rs are broadly tuned in terms of agonist activation. Some of the commonly used bitter tastants include chloroquine, quinine, denatonium, aristocholic acid, saccharine, and noscapine. We investigated the effect of two TAS2R agonists, chloroquine and quinine on asthma in murine models. The receptor subtype specificity of these two bitter tastants has been established using a heterologous expression system^22^. Chloroquine and quinine bind to TAS2R3, 7, 10, and 39, and TAS2R4, 7, 10, 14, 39, 40, 43, 44 and 46, respectively. Although TAS2R expression is found on multiple resident airway cells and on immune cells, the subtype of TAS2Rs expressed on different types of cells is different. Using broad-tuned ligands allowed us to discern the effect of bitter tastants on multiple features of asthma pathogenesis in mice.

TAS2R expression was demonstrated in human leukocytes primarily on lymphocytes (11 different subtypes with 3 of them being expressed at significantly high levels in asthmatics) and mast cells (9 different subtypes). Stimulation of blood leukocytes with TAS2R agonists resulted in inhibition of LPS-induced release of IL-13, IL-4, IL-5, TNF-α, IL-1β and IFN-γ, all of which are known to play an important role in asthma^23^. TAS2R agonists chloroquine and denatonium inhibited IgE-induced activation of mast cells and degranulation reflected by the inhibition of release of PGD2 and histamine^24^. Mast cell activation is known to contribute to asthma pathogenesis and AHR. These two studies demonstrate the potential anti-inflammatory effect mediated *via* activation of TAS2Rs on immune cells. Using two different murine models of allergic asthma, we demonstrate significant and clinically relevant inhibition of immune cell infiltration as well cytokines/chemokines release in the lung by TAS2R agonists. Our findings also demonstrate a differential regulation of cytokines and chemokines release by HDM and OVA in mice. Of note, the anti-inflammatory effects of TAS2R agonists have been demonstrated in the upper airways and respiratory epithelium in chronic rhinosinusitis^25^,^26^. More importantly our studies using both animal models of asthma, and mechanistic studies in humans *in vitro* clearly demonstrate that TAS2R agonists dampen global allergen-induced airway inflammation and have broader application in terms of reducing dependence on inhaled steroids, mainstay maintenance therapy for asthma.

A genomics study recently demonstrated that TAS2R expression is increased in peripheral blood leukocytes, and the expression level of TAS2R is correlated with severity of the disease in asthmatics^23^. Should such an upregulation of TAS2R with asthma extend to other cells/tissues including ASM, this disease feature would further favor the use of TAS2R agonists as asthma therapeutics given receptor upregulation would help protect against receptor desensitization which is believed to limit the therapeutic efficacy of anti-asthma GPCR agonists, including the beta-2-adrenoceptor^27^,^28^.

Our findings in both the HDM and OVA models suggest a significant inhibition of mucus accumulation in the airways when treated with TAS2R agonists. Shah *et al*.^29^ demonstrated that TAS2Rs are expressed on ciliary epithelium of airways and stimulation with bitter tastants results in increased ciliary beat frequency and hence might hasten mucus clearance. Gene expression data from total RNA isolated from murine lung revealed inhibition of allergen-induced upregulation of MUC-5 gene by TAS2R ligands. Therefore, inhibition of mucus synthesis and enhancement of clearance represent a means by which bitter tastants mediate the inhibition of mucus accumulation in the lung we observed herein on two different mouse models.

Our results demonstrate that TAS2R agonists inhibit allergen-induced airway remodeling characterized by deposition of extracellular matrix proteins such as collagen and fibronectin. Interestingly, ASM mass was also decreased in the animals treated with TAS2R agonists. A recent study from our laboratory demonstrated that TAS2R agonists chloroquine, quinine and saccharine inhibit mitogen-induced growth of cultured human ASM cells in a dose-dependent fashion^21^. These observations are valuable considering the fact the current anti-asthma medications are effective in either inhibiting airway inflammation or reversing bronchoconstriction, but have limited or no utility in treating airway remodeling, a important clinical feature in chronic asthmatics^5^,^6^,^7^,^30^. ASM mass is directly correlated to asthma symptoms in humans^31^,^32^.

Beta-agonists, the mainstay rescue medications in asthma, modestly inhibit ASM growth *in vitro*. Activation of PKA is central to ASM relaxation and ASM cell growth inhibition by beta agonists^9^. TAS2R agonists on the other hand do not activate cAMP/PKA pathway, yet induce a robust bronchodilatory and anti-proliferative effect on airways^21^. These studies suggest TAS2R agonists activate novel signal transduction mechanisms in airways cells to promote anti-asthma effects. Allergen challenge in murine models resulted in the secretion of TGF-β, MMPs and activation of pro-fibrotic signaling (e.g. p-Smad); treating animals with TAS2R agonists inhibited all of these features. These findings suggest inhibition of gene expression by TAS2R agonists in airways cells. We recently demonstrated that the anti-mitogenic effect of TAS2R agonists on human ASM cells involves inhibition of gene expression required for cell cycle progression^21^.

Lung function measurements in allergen-challenged mice demonstrate the ability of TAS2R agonist pretreatment to inhibit the induction of AHR caused by either HDM or OVA. AHR occurs not only due to calcium sensitization of ASM during inflammation, but is also promoted by structural and functional changes in airway cells upon allergen exposure. Herein we demonstrate that not only do TAS2Rs function as effective acute bronchodilators, but can also mitigate features of allergen-induced airway inflammation and remodeling that contribute to AHR.

In summary, our studies using two different murine model of asthma demonstrate that TAS2R agonists chloroquine and quinine administered by aerosol or intranasal route inhibit allergen-induced airway inflammation, remodeling, mucus production and AHR, four cardinal features of human asthma. TAS2Rs are known to be ubiquitously expressed and regulate a variety of cellular functions. Although several natural and synthetic compounds with known pharmacological characteristics are known to activate TAS2Rs, future studies are needed to develop TAS2R agonists and antagonists with high affinity and high subtype specificity. Alternately, existing drugs could be repurposed for asthma therapy. Advances in medicinal chemistry and computational modeling should catalyze the drug discovery process in exploiting TAS2Rs as novel anti-asthma therapeutic target.

## Materials and Methods

### Chemicals and reagents

Alexa Fluor-conjugated secondary antibodies were obtained from Life Technologies (Carlsbad, CA), IRDye-conjugated anti-mouse and anti-rabbit secondary antibodies were obtained from LI-COR Biosciences (Lincoln, NE). Mouse anti-smMHC, anti-sm-α-actin, anti-calponin, rabbit anti-fibronectin were obtained from Sigma (St. Louis, MO) and anti-phospho Smad2 and anti-Smad2 antibodies were purchased from Cell Signaling (Danvers, MA). Sircol collagen assay was obtained from Biocolor Life Sciences, UK. Staining kits (PAS, Trichrome), chloroquine, quinine and saccharine were obtained from Sigma. H&E and Afog staining kits were obtained from Vector Labs. All other chemicals were of analytical grade.

### Human volunteer recruitment

The study was approved by the Human Research Ethics Committee, The University of Sydney prior to commencement. All methods were performed in accordance with the relevant guidelines and regulations of the institution. Participants were required to be over 18 years of age. Exclusion criteria included if they were asthmatic, pregnant, known to faint during venipuncture procedures or had a bloodborne infection or condition. All patients provided written informed consent, and basic demographic information was collected.

### Mouse models of allergic asthma

All animal procedures were approved by the Institutional Animal Care Committee of Thomas Jefferson University and University of Maryland, Baltimore. All methods were performed in accordance with the relevant guidelines and regulations of the institutions. Animal surgeries were performed under tribromoethanol (Avertin, 250 mg/kg) anesthesia.

#### Prophylactic model

Female BALB/c mice at 8 weeks were intranasally challenged five days a week for three consecutive weeks with 25 μg of house dust mite (HDM) extract (*Dermatophagoides pteronyssinus*, Greer Labs, USA) in 35 μl saline (Fig. 1A). A select set of mice were administered either chloroquine (50 mg/kg), quinine (10 mg/kg) or vehicle (1% ethanol) in 25 μl volume by intranasal route 30 min prior to the HDM challenges. Similarly, mice were sensitized by injecting 2 mg alum in 0.2 ml PBS or alum with 100 μg ovalbumin (OVA) per mouse on day 1 and day 14 intraperitoneally (Fig. 1B). Mice were then challenged with aerosolized sterile PBS with or without 1% OVA on days 19, 21, 23, 25 and 27 for 30 min. Select sets of animals were treated with 1.5 mg/ml chloroquine or quinine for 30 min by aerosol prior to OVA challenge. Twenty-four hours after the last challenge, lung function measurements were performed, bronchoalveolar lavage (BAL) fluid was collected, fresh or formalin-fixed lungs were harvested.

#### Treatment model

Female BALB/c mice (8 weeks old) were initially challenged 5 days a week for 3 weeks via intranasal administration of 25 μg of HDM extract in 35 μl saline. Following this, mice were treated with chloroquine (50 mg/kg), quinine (10 mg/kg) or vehicle (1% ethanol) in 25 μl volume by intranasal route 30 min prior to subsequent HDM challenges for additional 2 weeks. Twenty-four hours following the last challenge, lung function measurements were performed, bronchoalveolar lavage (BAL) fluid was collected and fresh or formalin-fixed lungs were harvested for downstream analyses.

### Aerosol characterization and quantification

Chloroquine was dissolved in water to a concentration of 1.5 mg/ml. Drug was nebulized by an AirLife Misty Max 10 nebulizer with 10 liter/min of air and captured by an Anderson Cascade Impactor (3 M) or glass impinger (Ace) according to standard practice. Collected samples and standards were then separated on a Luna 3 u Phenyl-Hexyl column (Phenomenex), peaks were ionized (Acquity TQD) and analyzed using Mass Lynx (v4.1).

### Assessment of BAL cellularity

BAL samples were subjected to centrifugation and cell pellet resuspended in 1 ml PBS. Total cell count in BAL samples was determined by hemocytometer and data expressed as cells/ml. The BAL cells after centrifugation were stained with Hema-3 staining kit (Fisher Scientific) and differential cell count was determined by brightfield microscope.

### Measurement of BAL cytokines and chemokines

The content of cytokines and chemokines in BAL fluids were measured by Multiplexing LASER Bead Technology (Eve Technologies, Calgary, Canada) using a 31-Plex-mouse cytokine/chemokine array (Cat #: MD31) and expressed as pg/ml.

### Preparation of Lung Lysates

Mouse lungs were cut into small pieces in 250 μl of lysis buffer (40 mM Tris, 150 mM NaCl, 1% IgepalCA-630, 1% deoxycholic acid, 1 mM NaF, 5 mM β-glycerophosphate, 1 mM Na_3_VO_4_, 10 μg/ml aprotinin, 10 μg/ml leupeptin, 7 μg/ml pepstatin A, 1 mM PMSF, pH 8.0). Lung tissues were homogenized using a polytron. The lysate was centrifuged (760 × *g*, 5 min) and the supernatant stored at −80 °C for subsequent analyses.

### Immunoblotting

Protein concentration was determined by Pierce BCA assay kit (Thermo Scientific, Rockford, IL) and subjected to immunoblot analysis using protocols described previously^10^,^33^ with primary antibodies noted above (1:1000), followed by incubation with respective secondary antibodies (LI-COR Biosciences) (1:5000). Immunoblots were visualized and bands quantified using the Odyssey infrared imaging system (LI-COR Biosciences).

### Soluble Collagen Assay

Total soluble collagen content in the lung lysates was assessed using Sircol collagen assay (Biocolor, UK) according to the manufacturer’s protocol as per^34^. Collagen assay was performed by mixing lung homogenates with Sircol Dye reagent and measuring absorbance using a plate reader. Collagen content was quantified using a standard curve generated by reference standards and was normalized to the total lung protein content in each sample.

### Histopathological analysis

Paraffin embedded tissues were used for histological evaluation as described previously^33^,^35^,^36^,^37^, using 5 μm sections mounted on Superfrost Plus slides. Slides were deparaffinized, rehydrated and subjected to heat-induced epitope retrieval. The sections after blocking were stained with the primary antibody (1:100 anti-sm-α-actin) at 4 °C overnight followed by staining with 1:250 goat anti-mouse Alexa Fluor conjugated secondary antibody at room temperature for 1 hour. The sections were washed in PBS and stained with Draq5 for 15 minutes. Finally, the sections were washed, mounted with Prolong Antifade (Molecular Probes, USA), and fluorescent imaging performed using an Olympus BX-51 fluorescent microscope. The lung tissues fixed in 10% formalin, embedded in paraffin were cut and stained with H&E, trichrome blue, AFOG and PAS staining using a standard histological protocol^36^. Image acquisition and analysis was performed using a brightfield microscope.

Lung inflammatory changes were graded using a semi-quantitative scoring system based on the following parameters: peribronchial inflammation and degree of total cell infiltration. Peribronchial inflammation was graded on a scale of 0–4 with 0, absent; 1, slight; 2, mild; 3, moderate; and 4, severe. The degree of cell infiltration was scored of 0–3 with 0, no cells; 1, few cells; 2, moderate influx of cells; and 3, extensive influx of cells. The total lung inflammation score was expressed as the sum of the scores for each parameter.

Similarly, airway fibrosis in the formalin-fixed lung tissue was analyzed using AFOG or Trichrome stain and scored by using a scoring system for measuring subepithelial fibrosis (increased content of ECM protein such as collagen, stains blue with AFOG and Trichrome). A scoring range of 0–3 was used with 0, none; 1, infrequent; 2, common; and 3, widespread; blue staining in the airway wall.

### Peripheral blood neutrophil isolation and migration assay

Neutrophils were isolated from peripheral blood collected from volunteers as previously described^38^,^39^,^40^. Briefly, 40 ml of blood was mixed with 10 ml acid citrate dextrose (ACD), 10 ml of phosphate buffered saline (PBS) (Gibco, Carlsbad, CA, USA), and 6 ml of 10% dextran (MP Biomedicals, Santa Ana, USA) and left for 20 minutes for sedimentation to occur at room temperature. The top layer was removed, overlaid on Ficoll Paque-PLUS (GE Healthcare, Little Chalfont, UK), and centrifuged at 490 g for 10 minutes. The supernatant was discarded and the cell pellet of granulocytes was resuspended in sterile water for 30 seconds to lyse remaining red blood cells before osmolarity was reestablished with equal parts of 2x PBS. Cells were then incubated for 30 minutes at 4 °C with CD16 magnetic beads (Miltenyi Biotec, Bergisch, Germany) before running through a magnetic column as per the manufacturer’s instructions. Previous optimization of the protocol showed typical purity was 99% or greater by a haematoxylin and eosin stain. Neutrophils were resuspended in 1% FBS, 1% 1 M HEPES, and 1% penicillin/streptomycin RPMI 1640 at 5 × 10^6^ cells/ml and were co-incubated (250,000 cells/well) with TAS2R agonists (chloroquine and quinine; 100 μM, 500 μM and 1 mM) in the upper well of the Transwell inserts (Corning, Corning, New York, USA) while CXCL8 (100 ng/ml, R&D Biosciences, USA) was added to stimulate cell chemotaxis in the lower well. Following an incubation period (90 min at 37 °C, 5% CO_2_), the cells that have migrated through the membrane were stained with trypan blue and manually counted.

### Measurement of Lung Mechanics

Lung mechanics was measured using a flexiVent system (Scireq, Canada) as described previously^10^,^35^,^36^,^41^. Anesthetized and intubated mouse was ventilated with a tidal volume of 250 μl at 150 breaths/minute. A PEEP of 3 cm H_2_O was used for all studies. Mice were subjected to an increased dose of nebulized methacholine (MCh) challenge protocol using an in-line ultrasonic nebulizer. Using low frequency forced oscillation technique, respiratory mechanical input impedance (*Zrs*) was derived from the displacement of the ventilator’s piston and the pressure in its cylinder. By fitting *Zrs* to the constant phase model, flexiVent software calculated airway resistance (R), which was normalized to body weight. Change in lung resistance (R) was recorded for each dose of MCh. Animals were treated with aerosolized chloroquine (250 μg/ml) or quinine (150 μg/ml) at the end of MCh challenge and change in lung resistance recorded.

### Data analyses

Values reported for all data represent means ± standard error of means (SEM). For all studies, replicate data from at least 8–11 mice were obtained. The statistical significance of differences between two means was determined by one-way or two-way ANOVA with Bonferroni’s multiple comparison test for comparison between treatments or Tukey’s multiple comparison test. Differences were considered to be statistically significant when *p* < 0.05.

## Additional Information

**How to cite this article**: Sharma, P. *et al*. Bitter Taste Receptor Agonists Mitigate Features of Allergic Asthma in Mice. *Sci. Rep.* **7**, 46166; doi: 10.1038/srep46166 (2017).

**Publisher's note:** Springer Nature remains neutral with regard to jurisdictional claims in published maps and institutional affiliations.

## Figures and Tables

**Table 1:** Differential cell counts (prophylactic model and treatment model).

Allergen-induced asthma models	Differential BALF counts (cell numbers ± SEM)			
	Eosinophils	Neutrophils	Macrophages	Lymphocytes
**Prophylactic model**				
*Allergen – HDM*				
Saline control	329 ± 170	763 ± 283	60,725 ± 13,721	21,550 ± 4,800
Vehicle + HDM	301,750 ± 22,605^*^	227,583 ± 16,484^*^	242,250 ± 20,579^*^	131,450 ± 20,437^*^
CQ + HDM	151,909 ± 17,391^#^	153,655 ± 17,520^#^	168,227 ± 18,120^#^	101,936 ± 13,797^#^
Q + HDM	121,778 ± 6,984^#^	123,133 ± 12,419^#^	150,778 ± 17,505^#^	88,689 ± 15,446^#^
*Allergen - Ovalbumin*				
Saline control	129 ± 129	2,137 ± 966	164,888 ± 63,857	71,250 ± 25,706
Vehicle + OVA	228,700 ± 40,250^*^	217,900 ± 17,124*	258,200 ± 34,905	117,590 ± 12,947
CQ + OVA	114,430 ± 6,335^#^	113,030 ± 6,824^#^	208,080 ± 37,431	67,120 ± 8,401
Q + OVA	134,900 ± 21,007^#^	147,330 ± 20,739^#^	171,660 ± 29,377	98,680 ± 19,500
**Treatment model**				
	*Allergen - HDM*			
Saline control	353 ± 234	1079 ± 397	95,911 ± 9,907	39,700 ± 3,994
Vehicle + HDM	222,625 ± 18,383^*^	274,500 ± 36,029^*^	302,250 ± 29,842^*^	149,438 ± 17,656^*^
CQ + HDM	147,300 ± 10,964^#^	177,000 ± 14,052^#^	240,875 ± 20,747	130,750 ± 17,465
Q + HDM	149,000 ± 19,431^#^	149,375 ± 15,427^#^	203,750 ± 17,783^#^	154,683 ± 27,903

Data shown above represents the mean of 8–11 animals in each group; ^*^p < 0.05 (Saline vs HDM or OVA), ^#^*p* < 0.05 (HDM or OVA vs CQ or Q) using 1-way ANOVA. CQ: chloroquine; Q: quinine; OVA: Ovalbumin; HDM: house dust mite; BALF: bronchoalveolar lavage fluid; SEM: standard error of means.

**Table 2:** BALF Cytokines and chemokines release in prophylactic models.

Cytokines/Chemokines (pg/ml ± SEM)	Treatment groups							
	*Allergen - HDM*				*Allergen – OVA*			
	Saline control	Vehicle + HDM	CQ + HDM	Q + HDM	Saline control	Vehicle + HDM	CQ + HDM	Q + HDM
IL-4	0.16 ± 0.04	14.68 ± 3.70^*^	7.15 ± 0.90^#^	6.47 ± 1.27^#^	0.21 ± 0.06	12.02 ± 1.92^*^	4.97 ± 0.65^#^	6.76 ± 1.69^#^
IL-5	0.47 ± 0.07	34.22 ± 7.06^*^	15.97 ± 3.92^#^	18.64 ± 4.61^#^	0.53 ± 0.09	52.65 ± 6.44^*^	25.96 ± 6.64^#^	19.41 ± 5.67^#^
IL-9	11.64 ± 4.25	37.56 ± 7.53^*^	30.25 ± 6.48	17.50 ± 6.36^#^	2.28 ± 0.42	13.08 ± 9.44	10.33 ± 7.80	28.72 ± 11.73
IL-13	0.11 ± 0.06	19.67 ± 3.35^*^	7.83 ± 2.50^#^	4.40 ± 1.34^#^	0.12 ± 0.08	14.32 ± 1.48^*^	3.25 ± 0.85^#^	6.12 ± 2.56^#^
IL-17	0.15 ± 0.05	1.26 ± 0.25^*^	0.82 ± 0.22	0.84 ± 0.21	0.11 ± 0.05	1.16 ± 0.16^*^	0.50 ± 0.14^#^	0.65 ± 0.17^#^
IL-10	0.48 ± 0.14	66.44 ± 26.52^*^	21.15 ± 7.81	2.64 ± 0.54^#^	n.d.			
Eotaxin	10.54 ± 2.01	98.19 ± 14.78^*^	62.45 ± 7.89^#^	39.20 ± 9.41^#^	13.82 ± 5.31	147.30 ± 43.11^*^	53.29 ± 11.17^#^	54.65 ± 10.43^#^
KC	19.5 ± 1.91	483.80 ± 114.20^*^	219.90 ± 56.88^#^	196.70 ± 63.07^#^	36.77 ± 3.79	766.00 ± 50.93^*^	480.30 ± 81.20^#^	501.00 ± 52.83^#^
TNF-α	1.24 ± 0.43	18.90 ± 3.41^*^	10.90 ± 2.09^#^	6.72 ± 1.36^#^	0.81 ± 0.35	6.61 ± 1.18^*^	4.14 ± 0.77	4.98 ± 0.67
IP-10	3.48 ± 0.40	26.32 ± 4.80^*^	18.58 ± 2.42	15.63 ± 2.50^#^	4.48 ± 1.07	20.02 ± 3.36^*^	14.44 ± 2.04	14.16 ± 1.61
RANTES	0.14 ± 0.12	8.67 ± 2.30^*^	3.40 ± 0.80^#^	2.12 ± 0.42^#^	0.31 ± 0.20	2.99 ± 0.83^*^	1.23 ± 0.39	1.86 ± 0.26

Data shown above represents the mean of 8–11 animals in each group; ^*^p<0.05 (Saline vs HDM or OVA), ^#^*p* < 0.05 (HDM or OVA vs CQ or Q) using 1-way ANOVA. CQ: chloroquine; Q: quinine; OVA: Ovalbumin; HDM: house dust mite extract; SEM: standard error of means; KC: keratinocyte chemoattractant; IP-10: interferon-γ-induced protein 10; RANTES: regulated on activation, normal T cell expressed and secreted; n.d.: not detected.

**Table 3:** Treatment model for HDM-induced allergic airway disease.

Cytokines/Chemokines (pg/ml ± SEM)	Treatment groups (Allergen – HDM)			
	Saline control	Vehicle + HDM	CQ + HDM	Q + HDM
Eotaxin	10.39 ± 1.06	66.45 ± 14.91^*^	37.53 ± 5.71^#^	25.73 ± 5.23^#^
KC	32.58 ± 3.43	183.20 ± 33.34^*^	105.10 ± 19.25^#^	111.30 ± 14.34^#^
IP-10	4.32 ± 0.33	16.49 ± 3.26^*^	8.80 ± 1.59^#^	8.36 ± 1.33^#^
RANTES	4.98 ± 0.50	11.93 ± 2.24^*^	5.70 ± 0.74^#^	5.10 ± 0.68^#^
TGF-β1	47.48 ± 6.21	2518.00 ± 279.50^*^	1681.00 ± 220.50^#^	1552.00 ± 165.50^#^

Data shown above represents the mean of 8–11 animals in each group; ^*^p<0.05 (Saline vs HDM), ^#^*p*<0.05 (HDM vs CQ or Q) using 1-way ANOVA. CQ: chloroquine; Q: quinine; HDM: house dust mite extract; SEM: standard error of means; KC: keratinocyte chemoattractant; IP-10: interferon-γ-induced protein 10; RANTES: regulated on activation, normal T cell expressed and secreted; TGF-β1: transforming growth factor beta 1.
